# Decreased Intrinsic Neural Timescales in Mesial Temporal Lobe Epilepsy

**DOI:** 10.3389/fnhum.2021.772365

**Published:** 2021-12-08

**Authors:** Kefan Wang, Xiaonan Zhang, Chengru Song, Keran Ma, Man Bai, Ruiping Zheng, Yarui Wei, Jingli Chen, Jingliang Cheng, Yong Zhang, Shaoqiang Han

**Affiliations:** ^1^Department of Magnetic Resonance Imaging, The First Affiliated Hospital of Zhengzhou University, Zhengzhou, China; ^2^Key Laboratory for Functional Magnetic Resonance Imaging and Molecular Imaging of Henan Province, Zhengzhou, China; ^3^Engineering Technology Research Center for Detection and Application of Brain Function of Henan Province, Zhengzhou, China; ^4^Engineering Research Center of Medical Imaging Intelligent Diagnosis and Treatment of Henan Province, Zhengzhou, China; ^5^Key Laboratory of Magnetic Resonance and Brain Function of Henan Province, Zhengzhou, China; ^6^Key Laboratory of Brain Function and Cognitive Magnetic Resonance Imaging of Zhengzhou, Zhengzhou, China; ^7^Key Laboratory of Imaging Intelligence Research Medicine of Henan Province, Zhengzhou, China

**Keywords:** mesial temporal lobe epilepsy, fMRI, intrinsic neural timescale, information processing, cognitive impairment and emotional comorbidities

## Abstract

It is well established that epilepsy is characterized by the destruction of the information capacity of brain network and the interference with information processing in regions outside the epileptogenic focus. However, the potential mechanism remains poorly understood. In the current study, we applied a recently proposed approach on the basis of resting-state fMRI data to measure altered local neural dynamics in mesial temporal lobe epilepsy (mTLE), which represents how long neural information is stored in a local brain area and reflect an ability of information integration. Using resting-state-fMRI data recorded from 36 subjects with mTLE and 36 healthy controls, we calculated the intrinsic neural timescales (INT) of neural signals by summing the positive magnitude of the autocorrelation of the resting-state brain activity. Compared to healthy controls, the INT values were significantly lower in patients in the right orbitofrontal cortices, right insula, and right posterior lobe of cerebellum. Whereas, we observed no statistically significant changes between patients with long- and short-term epilepsy duration or between left-mTLE and right-mTLE. Our study provides distinct insight into the brain abnormalities of mTLE from the perspective of the dynamics of the brain activity, highlighting the significant role of intrinsic timescale in understanding neurophysiological mechanisms. And we postulate that altered intrinsic timescales of neural signals in specific cortical brain areas may be the neurodynamic basis of cognitive impairment and emotional comorbidities in mTLE patients.

## Introduction

Mesial temporal lobe epilepsy (mTLE), frequently characterized by the pathological substrate of hippocampal sclerosis (HS), is the most common type of refractory focal epilepsy (Berg, 2008). Growing evidences suggest that patients with mTLE are affected by a variety of psychiatric comorbidity (Harden, 2002; Vazquez and Devinsky, 2003; Keezer et al., 2016) and cognitive problems (Allone et al., 2017). Considering the high morbidity, severe cognitive problems and poor quality of life, the neuropathological mechanisms underlying mTLE should be fully understood to facilitate the development of more effective treatment.

mTLE is now recognized as a brain network disorder involving a wide range of brain regions far beyond the medial temporal lobe associated with origin of epilepsy (Cataldi et al., 2013). Meanwhile, mTLE is characterized by transient abnormal electrical activity both during ictal and interictal period (Morgan et al., 2020). And interictal epileptic discharges (IEDs) have also been shown to affect neuronal activity in brain regions farther from the epileptogenic focus (Tong et al., 2019). It is well known that many high order brain areas such as prefrontal cortices, anterior cingulum, posterior lobe of cerebellum, and insular, have been demonstrated to be disrupted in mTLE by previous studies with multimodality medical image, for example, hypoperfusion (Blumenfeld et al., 2004), abnormal neural connectivity (Holmes et al., 2014; Dinkelacker et al., 2015; Burianová et al., 2017) and damaged structure (Bernasconi et al., 2004; Riederer et al., 2008; Santana et al., 2010). These brain regions abnormalities are associated with psychiatric (Seeley et al., 2007; Jiang et al., 2018) and cognitive symptoms (Holmes et al., 2014; Burianová et al., 2017) in mTLE. Recently, evidence also showed that the all-consuming and stereotypical epileptic discharge is associated with the destruction of the information capacity of the network and prevents normal information processing in the surrounding area (Trevelyan et al., 2013), suggesting a possible mechanism for cognitive impairment from a local neurodynamic perspective (Japaridze et al., 2014). However, the way in which the neurodynamics involved in information processing in these brain regions are disrupted remains unknown.

Recently, in order to get a deeper understanding of dynamics of local neural organization, Watanabe et al. (2019) proposed a novel approach on the basis of resting-state fMRI data to depict how long neural information is likely to be stored in a neural area, a fundamental functional property of the local brain region named intrinsic neural timescales (INT), by computing the positive magnitude of the autocorrelation of intrinsic neural signals in the local brain area. INT is considered to represent the time window of neural information integration and reflect an ability to accumulate information over a long period of time (Murray et al., 2014; Watanabe et al., 2019; Wengler et al., 2020). Just like the hierarchy of spatial receptive field sizes well established in the visual cortex (Murray et al., 2014), neural timescales have also been proposed to exhibit a brain hierarchy, that is, a gradient from fast (caudal) to slow (rostral) (Kiebel et al., 2008). Functional specialization across areas is associated with the appropriate neural timescales in the corresponding brain region (Murray et al., 2014): A longer INT is often found in higher-order brain regions, such as prefrontal areas, supporting stable cognitive processes which rely on long-term accumulation of information (Chen et al., 2015). In contrast, a shorter INT in sensory areas means more random activity (Watanabe et al., 2019), which facilitates rapid responses to changing stimuli in the environment (Chaudhuri et al., 2015). This new avenue has been successfully applied to investigate dynamics of local brain regions in the resting state in patients with autism spectrum disorders (ASDs) (Watanabe et al., 2019) and schizophrenia (Wengler et al., 2020). Specially, Watanabe et al. (2019) suggested faster intrinsic timescales in sensorimotor cortex showed a significant and reproducible correlate with atypical behavior in autism.

However, as of today, no study has investigated the alteration of INT through the newly developed method in mTLE patients. Considering that patients with epilepsy are characterized by impaired information processing (Trevelyan et al., 2013; Japaridze et al., 2014; Citherlet et al., 2020; Shuman et al., 2020), which is believed to be associated with common pathological processes of epilepsy and cognitive impairment (Japaridze et al., 2014), we hypothesized that intrinsic timescales of neural signals should be atypical in patients with unilateral mTLE.

In our current study, patients with unilateral mTLE were collected and compared with matched healthy controls (HC) to investigate abnormal INT in patients. In addition, we paid attention to whether the pattern of INT changes exists difference among patients with different duration of epilepsy or different side of the epileptogenic focus (HS).

## Materials and Methods

### Clinical Data

We recruited 36 patients with unilateral mTLE (14 left- and 22 right-side) as the epilepsy group who were consulting in the Neurology Clinic and Inpatient Department of The First Affiliated Hospital of Zhengzhou University and 36 healthy volunteers as the control group. The diagnosis was based on the detailed history, neurological examination, electroencephalography (EEG) recordings, and imaging findings. Patients (22 females, 29.08 ± 10.12 years of age) who fulfilled the following criteria were included: (1) described or observed clinical semiology consistent with seizures of mesial temporal lobe origin; (2) MRI results that suggested hippocampal sclerosis of the patients; (3) unilateral interictal and ictal epileptic discharges according to scalp EEG or intracranial electrode EEG; (4) all right-handed. Exclusion criteria for the patient group are as follows: (1) patients with brain structural abnormalities (except for hippocampal sclerosis), other systemic diseases, psychiatric disorder, history of alcohol abuse, etc.; (2) Un-identified lateralization of mTLE; (3) head motion during scanning is greater than 3 mm.

Inclusive criteria for the control group (23 females, 28.61 ± 9.72 years of age) are as follows: (1) normal head MRI results; (2) with at least primary school education; (3) all right-handed. Exclusion criteria: Patients with any prior history of neurological or psychiatric diseases. Handedness was rated according to the Edinburgh Handedness Inventory.

This study was approved by the research ethical committee of The First Affiliated Hospital of Zhengzhou University, and informed consent was obtained from all subjects.

### MR Imaging Acquisition and Preprocessing

Resting-state fMRI data were acquired using a 3 T Magnetom Prisma MRI scanner (Siemens Healthcare, Erlangen, Germany) with a 64-channel head coil. All subjects were told to lie down flat, not think, open their eyes, and breathe quietly. The scanning parameters were as follows: TR/TE, 1,000/30 ms, FOV, 220 × 220 mm^2^, slice thickness, 2.2 mm, slice gap, 0.4 mm, flip angle, 70°, and voxel size, 2.0 × 2.0 × 2.2 mm^3^, with 52 slices and 400 dynamics.

The Data Processing and Analysis of Brain Imaging (DPABI) toolbox^1^ was used to preprocess the statistics. The following steps were performed: (1) conversion of data formats; (2) removal of the first 10 time points; (3) slice-timing correction; (4) realignment; Participants were excluded if their maximal head motion exceeded 3 mm displacement or 3°of rotation. No subjects were excluded after that. (5) spatial normalization of fMRI images to the standard EPI template and resampled to 3 × 3 × 3 mm^3^ resolution; (6) detrending; (7) regression of the Friston-24 motion parameters (Satterthwaite et al., 2012), cerebrospinal fluid signal, white matter signal, and global-brain signal; (8) band-pass temporal filtering (0.01–0.08 Hz); (9) calculation of the mean frame-wise displacement (FD) of each subject to assess the head movement (Power et al., 2012; Zhang et al., 2017); There was no significant difference of mean FD between the mTLE and HC groups (*p* = 0.159 in a Mann–Whitney *U* test). (10) scrubbing with cubic spline interpolation to remove the “bad” time points and their 1-back and 2-after time points on the basis of FD threshold of 0.5 mm; (11) spatial smoothing with a 6 mm full-width-at-half-maximum Gaussian kernel to improve the signal-to-noise ratio (SNR).

### Intrinsic Neural Timescale Maps

According to the steps described previously (Watanabe et al., 2019; Wengler et al., 2020), the intrinsic timescale of each voxel was estimated using the preprocessed fMRI data at the level of a single participant.

First, for each voxel, an autocorrelation function (ACF) of fMRI signals [time bin = repetition time (TR)] was estimated. Next, we calculated the sum of ACF values during the initial positive period. This initial positive period begins with the first lag and ends at the time point just before the first timepoint with a non-positive autocorrelation coefficient. Then the sum of ACF values was multiplied by the TR of the fMRI data to adjust for differences in temporal resolution. The above steps were achieved through the custom MATLAB code publicly available at https://github.com/RaichleLab. Finally, this final brain map was used as an intrinsic neural timescale map.

### Statistical Analysis

For demographic and clinical characteristics, differences in age and gender between the group of mTLE and HC were analyzed with two-sample *t* test and Chi-square test, respectively (*P* < 0.05). Differences in age and mean FD among subgroup I, subgroup II, and HC were analyzed with one-way analysis of variance (ANOVA) and Kruskal–Wallis ANOVA, respectively (*P* < 0.05). Difference in gender among subgroup I, subgroup II, and HC was analyzed with Chi-square test (*P* < 0.05). Difference in lateralization between subgroup I and subgroup II was analyzed with Chi-square test (*P* < 0.05). Differences in age and illness duration between the subgroups of LTLE and RMTLE were analyzed with two-sample *t* test (*P* < 0.05). Differences in gender and mean FD between the subgroups of LTLE and RMTLE were analyzed with Chi-square test and Mann–Whitney *U*-test, respectively (*P* < 0.05).

First, two-tailed two-sample *t* test was performed on the timescale maps using SPM12 toolkit to determine regions showing significantly altered INT between groups of mTLE and HC. Multiple comparison correction was performed using a Gaussian random field (GRF) where threshold of voxel-wise *p* < 0.001 and cluster-level *p* < 0.05 [the cluster-level correction refers to familywise error rate (FEW)] in DPABI toolbox (we set cluster extent threshold at 30 voxels).

Secondly, we divided patients into two subgroups according the duration of the epilepsy (subgroup I: *n* = 17, epilepsy duration<10 years, subgroup II: *n* = 19, epilepsy duration ≥ 10 years). ANOVA was used to detect the differences among the three groups (subgroup I, subgroup II, and HC) (GRF corrected, *P*_voxel_ < 0.001, *P*_cluster_ < 0.05). The ANOVA result map was applied as mask to perform secondary analysis. Then pairwise between-group differences were investigated by two-sample *t* test (GRF corrected, *P*_voxel_ < 0.001, *P*_cluster_ < 0.05).

Thirdly, according to the side of the epileptogenic focus, patients were divided into two subgroups: the left mTLE group (L-mTLE) and the right mTLE group (R-mTLE). We used two-sample *t* test to compare INT maps between the two subgroups (GRF corrected, *P*_voxel_ < 0.001, *P*_cluster_ < 0.05).

In all above comparisons, age, gender and mean FD were treated as nuisance covariates.

### Assessment of Robustness

To rule out the effect of head motion and the quality of the imaging data on our results, we calculated the correlation of mean FD and SNR (Welvaert and Rosseel, 2013) with the altered INT values in patients with mTLE. The altered INT was defined as the mean values of peak coordinates with a spherical radius of 6 mm in brain regions with significant between-group differences. The peak coordinates of the decreased INT were in Table 2. A statistically significant threshold of *p* < 0.05 was set for all correlation analyses.

**TABLE 1:** Demographic and clinical information of subjects.

Characteristics	mTLE (*n* = 36)	HC (*n* = 36)	*P* value
Age (years)	29.08 ± 10.12	28.61 ± 9.72	0.841^a^
Sex (female: male)	22:14	23:13	0.808^b^
Duration (years)	11.10 ± 8.46	—	—
Lateralization (L:R)	14:22	—	—
mean FD (mm)	0.14 ± 0.06	0.13 ± 0.06	0.159^c^
SNR (dB)	82.46 ± 16.25	85.39 ± 14.00	0.415^a^

*All values are mean ± standard deviation.*

*L, left; R, right; FD, frame-wise displacement; SNR, signal to noise ratio; ^a^two-sample t test; ^b^Chi-square test; ^c^Mann–Whitney U-test;*

**TABLE 2:** Brain regions showing significantly decreased INT between mTLE patients and HC subjects (GRF corrected, *P*_voxel_ < 0.001, *P*_cluster_ < 0.05, voxel wise ≥ 30).

Regions	MNI coordinates			Voxels	*T* value
	*x*	*y*	*z*		
Inferior OFG_R	51	30	−15	73	−4.55
Middle OFG_R	39	54	−15	43	−5.40
Insula_R	33	24	0	30	−4.31
Cerebellar crus1_R	27	−78	−24	100	−5.28
Cerebellar crus2_R	39	−66	−39	32	−4.49
Cerebellum 6_R	30	−63	−21	85	−5.22

*INT, intrinsic neural timescales; mTLE, mesial temporal lobe epilepsy; HC, healthy control; MNI, Montreal Neurological Institute; L, left; R, right; OFG, orbital frontal gyrus.*

## Results

### Demographic and Clinical Information

There were no significant differences in age and gender between the group of mTLE and HC (*p* > 0.05) (Table 1). In subgroup analyses, there were no significant differences among the three groups (subgroup I, subgroup II, and HC) for age and gender (*p* > 0.05), and there was no statistical difference in side of epileptogenic focus between subgroup I and subgroup II (*p* > 0.05) (Supplementary Table 1). And there were no significant differences between the subgroups of LTLE and RTLE for age, gender, and illness duration (*p* > 0.05) (Supplementary Table 2).

### The Landscape of Intrinsic Timescales and Patterns of Intrinsic Neural Timescales Changes in mTLE

**FIGURE 1:**
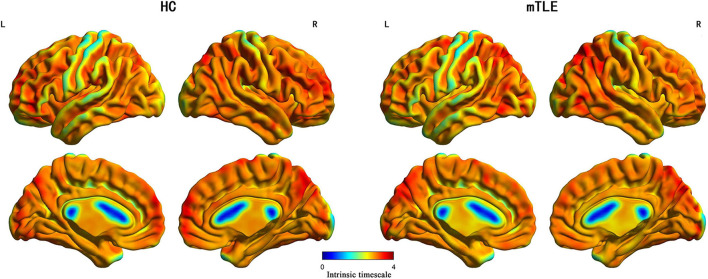
The spatial distribution maps of INT in both HC and mTLE groups.

The spatial distribution maps of INT for each group are shown in Figure 1. Longer timescales were highly localized in frontal and parietal cortices and shorter timescales in sensorimotor, visual, and auditory areas. Intrinsic timescales showed a similar whole-brain pattern of distribution in patients and HC groups.

**FIGURE 2:**
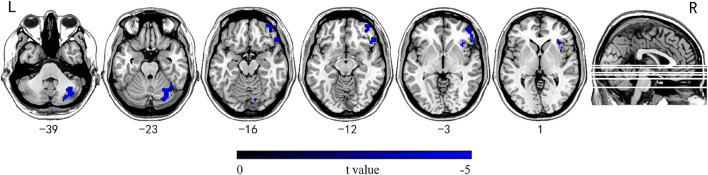
Brain regions showing significantly decreased INT between mTLE patients and HC subjects. Group differences in INT between the mTLE and HC groups were identified using a two-sample *t* test. The statistical significance level was set at voxel-wise *p* < 0.001, cluster-level *p* < 0.05 (GRF corrected, voxel wise ≥ 30). Patients with mTLE showed decreased INT in the right inferior/middle OFG, right insular, and right cerebellum crus1/crus2/6. INT, intrinsic neural timescales; HC, healthy control; mTLE, mesial temporal lobe epilepsy; OFG, orbital frontal gyrus; L, left; R, right.

The group comparison results of INTs revealed that patients with mTLE exhibited atypically shorter intrinsic timescales in the right inferior/middle orbital frontal gyrus (OFG), right insular, and right cerebellum crus1/crus2/6 (GRF corrected, *P*_voxel_ < 0.001, *P*_cluster_ < 0.05, voxel wise ≥ 30; Table 2 and Figure 2).

### Patterns of Intrinsic Neural Timescales Changes in mTLE Patients With Different Epilepsy Duration or Different Side of Epileptogenic Focus

**FIGURE 3:**
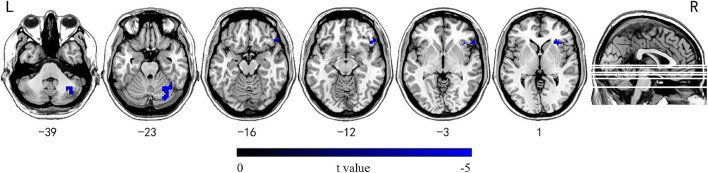
Brain regions showing significantly decreased INT between mTLE patients with longer epilepsy duration and HC subjects. Group differences in INT between the mTLE patients with longer epilepsy duration and HC groups were identified using a two-sample *t* test. The statistical significance level was set at voxel-wise *p* < 0.001, cluster-level *p* < 0.05 (GRF corrected, voxel wise ≥ 30). Patients with longer epilepsy duration showed decreased INT in the right inferior OFG and right cerebellum crus1/6. INT, intrinsic neural timescales; HC, healthy control; mTLE, mesial temporal lobe epilepsy; OFG, orbital frontal gyrus; L, left; R, right.

No significantly differences were detected in patients in subgroup I compared with HC or subgroup II. In subgroup II, the right inferior OFG and right cerebellum crus1/6 showed reduced INT compared with HC (GRF corrected, *P*_voxel_ < 0.001, *P*_cluster_ < 0.05, voxel wise ≥ 30; Figure 3 and Supplementary Table 3).

We observed no significantly differences between subgroups of L-mTLE and R-mTLE (GRF corrected, *P*_voxel_ < 0.001, *P*_cluster_ < 0.05).

### Results of Robustness Test

No significant correlation was observed between either mean FD or SNR and the altered INT values in mTLE patients (for all correlation analyses, *p* > 0.05). In addition, there was no significant difference between patients with mTLE and HCs either in mean FD (*p* = 0.159 in a Mann–Whitney *U* test) or in SNR (*p* = 0.415 in a two-sample *t* test) (Table 1).

## Discussion

In this study, the altered intrinsic timescale in mTLE patients was characterized for the first time. Patients with mTLE presented reduced intrinsic timescales in regions including right orbitofrontal cortex, insula, and cerebellum, implicating an impairment of information processing capability. Nevertheless, the pattern of INT changes showed no differences between patients with different stages or different side of the epileptogenic focus. These findings revealed altered local neural dynamics in mTLE and further promoted an integrative understanding of mTLE from the perspective of the dynamics of the neural activity.

### Atypical Intrinsic Neural Timescales in mTLE

Human cerebral cortex have been proven to follow a hierarchical ordering in intrinsic neural timescales (Murray et al., 2014; Demirtaş et al., 2019), and subcortical areas such as striatum, thalamus, and cerebellum also topographically mirrored this intrinsic timescale gradients (Raut et al., 2020). Consistent with previous findings, we revealed similar hierarchical timescales that longer timescales in frontal and parietal cortices and shorter timescales in sensorimotor, visual, and auditory areas. Given that INT is obtained by calculating the magnitude of autocorrelation of neural signals, the shorter intrinsic timescale in patients might indicates greater randomness and variability of brain signals (Watanabe et al., 2019). In the seizure propagation network, transient abnormal electrical activity may randomly disrupt functional connectivity within the network or with others with a consequently higher variability of brain signals (Morgan et al., 2015). Previous studies have found that patients with temporal lobe epilepsy performed atypically large signal variability in the prefrontal region (Laufs et al., 2014; Robinson et al., 2017; Tong et al., 2019) and insula (Tong et al., 2019), which mostly overlapping the regions showing decreased INT in our studies. Moreover, this temporal property in local brain signals was proposed to be in connection with the local gray matter volumes (GMVs) (Watanabe et al., 2019). Therefore, our study is supported by previous research which has revealed decreased GMVs in frontal lobe areas, limbic structures, and cerebellum in mTLE (Bernasconi et al., 2004; Riederer et al., 2008; Santana et al., 2010).

With regard to the shorter INT observed in mTLE patients both in our mainly analysis and subgroup analyses, it is unclear whether this arises from pathologic mechanisms directly related transient abnormal electrical activity (Morgan et al., 2020) or secondary changes of hippocampal sclerosis (Shuman et al., 2020). By developing a biophysical model, Chaudhuri et al. (2015) proposed that the INT hierarchy is implemented by a gradient of recurrent excitatory connections (excitatory-to-excitatory synapse strengths) that is scale by the hierarchal position of each region. Further, several studies demonstrated that imbalance of excitation-inhibition (E/I) ratio in individual brain regions could possibly result in aberrance of neural timescales (Kiebel et al., 2008; Hasson et al., 2015; Wengler et al., 2020), specially, elevated E/I ratio could lead to relative increases in neural timescales (Wengler et al., 2020). A review (Trevelyan et al., 2013) previously proposed that epileptic discharges almost completely inhibits all neuronal activity and then prevent normal processing by enhanced inhibition in surrounding areas, leading to disruption of normal information processing not only in ictal stage but also in interictal stage. Therefore, we suggest that the increases in the strength of recurrent inhibition in peripheral areas outside the seizure onset zone may lead to decreases in E/I ratio and further explaining reduced brain intrinsic timescale in our results. Besides, longer INT in cortical areas means greater ability to integrate and hold information, that is to say, new information can be maintained for a long time (Chaudhuri et al., 2015). Several studies have shown that hippocampal circuits contribute to continuous processing when they are without damage (Hannula et al., 2007; Olsen et al., 2012). And hippocampus has been reported to play an important role in the retainment of episodic memories (Hasson et al., 2015). A study in epileptic mice also showed that desynchronization of interneuronal firing between the CA1 and dentate gyrus (hippocampal subregion) significantly impacts spatial processing (Shuman et al., 2020). However, whether hippocampus is a necessary component during this process remains unclear. For instance, stimulus information in hippocampal amnesia can be retained long enough to participate in a conversation (Hasson et al., 2015), suggesting that information retention can be independent of the hippocampus. In our study, atypical INT was found in patients with mTLE whose hippocampus was structurally (Bernasconi et al., 2004) and functionally (Laufs et al., 2014) impaired, which to some extent may support the important role of hippocampus in information retention and processing in cortical areas.

Previous researches implicated that dysfunction in temporal lobe epilepsy was related to disease duration (Robinson et al., 2017), which supporting mTLE associated with hippocampal sclerosis is a progressive disorder (Zhang et al., 2017). In addition, lots of previous studies have provided evidence that heterogeneity have identified for L- mTLE and R-mTLE (Ridley et al., 2015; Lee et al., 2018). For example, there are different patterns of functional connection (Lee et al., 2018) and different degree of GMV atrophy (Riederer et al., 2008) between the left and right mTLE. Whereas, we observed no statistically significant changes between patients with long- and short-term epilepsy duration or between L-mTLE and R-mTLE. This might be due to the lack of sufficient sample size in our study.

### Decreased Intrinsic Neural Timescales in Higher-Order Regions and Its Effect on Cognitive or Emotional Comorbidities

mTLE showed decreased INT values in the insula, an area that constitutes the core region of the salience network (SN) (Menon and Uddin, 2010). The SN plays a role in the transition between default mode network (DMN) and central executive network (CEN) (Zhou et al., 2018), and consequently subserves the mediation of information flow across its interconnected brain networks involved in attentional processing and cognition (Menon and Uddin, 2010). As such, we considered that neurodynamics changes in the insular observed in our study may affect between-network interactions and ultimately higher cognitive functions which heavily rely on coordinated activity of resting state networks (RSNs) (Burianová et al., 2017). Moreover, SN is involved in maintaining consciousness and awareness (Burianová et al., 2017). Based on this, altered timescales in the salience network might account for the reduction or loss of awareness during seizures (Luo et al., 2014). Besides, decreased INT was also observed in OFC. The insular cortex and OFC are all belong to the affective network (AN), playing a broad role in modulating affective information (Li et al., 2018). Patients with temporal lobe epilepsy has been reported to have attentional bias for negative emotional information (Lanteaume et al., 2012). Given this, we hypothesize that decreased INT in the insular and OFC found in the mTLE group may potentially be one of neural bases of emotional lability, higher anxiety level and depression, which are common comorbidities in epilepsy (Harden, 2002; Vazquez and Devinsky, 2003; Keezer et al., 2016).

Right posterior lobe of cerebellum also exhibited decreased INT values. The posterior cerebellum, such as crus I/II which show robust structural (Kelly and Strick, 2003) and functional (Krienen and Buckner, 2009) correspondence with the prefrontal cortex, is engaged in intellect, social cognition and emotion control (Schmahmann, 2019). Posterior cerebellar impairments have been observed in patients with mTLE (Riederer et al., 2008). The abnormal INT values detected in present study might imply the disruption of neuronal activities in prefrontal-cerebellar circuits underpins the higher-level cognitive and affective deficits following cerebellar damage.

In our study, almost all brain regions with INT changes were higher order regions. Cognitive processes, such as decision-making (Cavanagh et al., 2016) and memory processing (Hasson et al., 2015), unfold over time and must therefore rely on the long-term accumulation and integration of information in those areas (Murray et al., 2014; Chaudhuri et al., 2015). Hence, significantly shorter INT in higher-level areas might lead to inefficient integration of information and this may point to the underlying neuropathology of cognitive or emotional impairments in mTLE.

### Robustness Test

We also focus on the effect of head motion and SNR on our results to a certain extent. The relationship between either mean FD or SNR and the altered INT values in patients with mTLE was not significant and there was no significant difference between mTLE and HCs either in mean FD or in SNR. It proved that the effect of head movement and SNR can be avoided in our study to some extent.

### Limitations

There are several limitations in the current study. First, the sample size is relatively small and all participants come from a single dataset. The main reason is that our inclusion criteria is very strict, only those TLE patients with unilateral hippocampal seizure lesions were enrolled. And the recruitment of healthy volunteers was performed using the matched control pairwise approach to increase the statistical power of these analyses. Future study could use another independent dataset and increase sample size to confirm our findings. Second, since there were no clear staging criteria for epilepsy progression, we only chose epilepsy duration for dividing the patients into two groups in the subgroup analysis, which obviously cannot represent the occurrence of epilepsy comprehensively. Third, potential effects of antiepileptic drugs in TLE patients were not considered. Finally, we did not calculate the correlation analysis between degree of hippocampal sclerosis and INT shortening and we will improve it in future studies.

## Conclusion

Our study revealed abnormal intrinsic timescale in patients with mTLE for the first time. Whereas, we found no statistically significant changes between patients with long- and short-term epilepsy duration or between left- and right-mTLE. This study provides new insight into the brain abnormalities of mTLE from the perspective of the dynamics of the brain activity, highlighting the significant role of intrinsic timescale in understanding the neurophysiological mechanisms. And we speculate that the reduced intrinsic timescales of neural signals in specific cortical brain regions might be the neurodynamic basis of cognitive deficits and emotional comorbidities in patients with mTLE.

## Data Availability Statement

The original contributions presented in the study are included in the article, further inquiries can be directed to the corresponding authors.

## Ethics Statement

The studies involving human participants were reviewed and approved by the Research Ethical Committee of The First Affiliated Hospital of Zhengzhou University. Written informed consent to participate in this study was provided by the participants’ legal guardian/next of kin.

## Author Contributions

JLiaC, YZ, and SH contributed to design of the study. KW, XZ, CS, KM, and MB collected the data. SH provided the methodological advice. KW and XZ performed data and statistical analysis. KW wrote the first draft of the manuscript. XZ, CS, KM, and RZ proofread the manuscript. YW and JLiC supervised the conduct of the study. All authors contributed to the article and approved the submitted version.

## Conflict of Interest

The authors declare that the research was conducted in the absence of any commercial or financial relationships that could be construed as a potential conflict of interest.

## Publisher’s Note

All claims expressed in this article are solely those of the authors and do not necessarily represent those of their affiliated organizations, or those of the publisher, the editors and the reviewers. Any product that may be evaluated in this article, or claim that may be made by its manufacturer, is not guaranteed or endorsed by the publisher.

## Footnotes

1
http://rfmri.org/dpabi
