# Supplementary material for: Decreased Intrinsic Neural Timescales in Mesial Temporal Lobe Epilepsy
Source: Front Hum Neurosci. 2021 Dec 8;15:772365. doi: 10.3389/fnhum.2021.772365 (PMC8693765; doi:10.3389/fnhum.2021.772365)
Supplement: Supplementary file 1 [file Data_Sheet_1.pdf]

Supplementary Material

1. Supplementary Tables

Supplementary Table 1. Demographic and clinical information of subjects in subgroups of short- and long- term epilepsy duration.

Characteristics	mTLE (n = 36)		HC (n = 36)	P value
	Subgroup I (epilepsy duration < 10years, n = 17)	Subgroup II (epilepsy duration ≥ 10years, n = 19)		
Age (years)	25.47 ± 10.78	32.32 ± 8.53	28.61 ± 9.72	0.112[a]
Sex (female: male)	9 : 8	13 : 6	23 : 13	0.614[b]
Lateralization (L:R)	7 : 10	7 : 12	—	0.79[b]
mean FD (mm)	0.16 ± 0.06	0.13 ±0.05	0.13 ± 0.06	0.09[c]

Abbreviations: All values are mean ± standard deviation. L = left; R = right; FD = frame-wise displacement. a One-way analysis of variance (ANOVA); b Chi-square test; c Kruskal-Wallis ANOVA;

Supplementary Table 2. Demographic and clinical information of subjects in subgroups of different side of epileptogenic focus.

Characteristics	mTLE (n = 36)		P value
	L-mTLE (n = 14)	R-mTLE (n = 22)	
Age (years)	27.93 ± 9.96	29.82 ± 10.39	0.592[a]
Sex (female: male)	10 : 4	12 : 10	0.311[b]
Duration (years)	10.65 ± 8.90	11.39 ± 8.36	0.800[a]
mean FD (mm)	0.13 ± 0.05	0.15 ± 0.06	0.180[c]

Abbreviations: All values are mean ± standard deviation. L = left; R = right; FD = frame-wise displacement; L-mTLE = left mesial temporal lobe epilepsy; R-mTLE = right mesial temporal lobe epilepsy. a two-sample t test; b Chi-square test; c Mann Whitney U-test;

Supplementary Table 3. Brain regions showing significantly decreased INT between mTLE patients with longer duration of epilepsy and HC subjects(GRF corrected, $P_{voxel}<0.001$, $P_{cluster}<0.05$, voxel wise≥30)

Regions	MNI coordinates			Voxels	*T* value
	x	*y*	*z*		
inferior OFG _R	45	24	-9	37	-5.75
cerebellar crus1_R	27	-78	-24	52	-5.40
cerebellum 6_R	30	-63	-24	63	-5.85

Abbreviations: INT = Intrinsic Neural Timescales; mTLE = mesial temporal lobe epilepsy; HC = healthy control; MNI = Montreal Neurological Institute; L=left; R=right; OFG = orbital frontal gyrus;
